# Microsporidiosis and Malnutrition in Children with Persistent Diarrhea, Uganda 

**DOI:** 10.3201/eid1501.071536

**Published:** 2009-01

**Authors:** Siobhan M. Mor, James K. Tumwine, Elena N. Naumova, Grace Ndeezi, Saul Tzipori

**Affiliations:** Tufts Cummings School of Veterinary Medicine, North Grafton, Massachusetts, USA (S.M. Mor, S. Tzipori); Tufts University School of Medicine, Boston, Massachusetts, USA (S.M. Mor, E.N. Naumova); Makerere University Medical School, Kampala, Uganda (J.K. Tumwine, G. Ndeezi)

**Keywords:** Enterocytozoon bieneusi, HIV/AIDS, Cryptosporidium spp., malnutrition, persistent diarrhea, children, Uganda, dispatch

## Abstract

We show that the microsporidian fungus *Enterocytozoon bieneusi* is associated with lower rates of weight gain in children in Uganda with persistent diarrhea. This relationship remained after controlling for HIV and concurrent cryptosporidiosis. Children with microsporidiosis were predicted to weigh 1.3 kg less than children without microsporidiosis at 5 years of age.

*Enterocytozoon bieneusi* is an important cause of persistent diarrhea, intestinal malabsorption, and wasting in HIV-positive adults. Mucosal damage associated with microsporidiosis is more extensive than that related to other opportunistic intestinal infections (*1**,**2*) and leads to substantial malabsorption of carbohydrates, fat, and essential nutrients (*2**–**5*). Although microsporidiosis is common in children <5 years of age, particularly those who live in developing countries (*6**,**7*) or who are HIV positive (*6**,**8**,**9*), the effects of infection on nutritional health of these vulnerable populations are not well documented. We reexamined anthropometric data of children in Uganda with persistent diarrhea (*6*) and used regression analysis to determine whether there is an association between microsporidiosis and reduced growth rates.

## The Study

A total of 243 children <60 months of age with persistent diarrhea (>14 days) were enrolled at Mulago Hospital in Kampala, Uganda, from November 2002 through May 2003. After informed consent was obtained, demographic, anthropometric, and clinical information was collected from each child. *E*. *bieneusi* spores were detected in stool specimens by using a nested PCR with *E*. *bieneusi*–specific primers (*7*). *Cryptosporidium* oocysts were detected by using immunofluorescence microscopy, with confirmation and genotyping subsequently determined by PCR–restriction fragment length polymorphism analysis (*10*). HIV status was determined by using established methods, and children positive for HIV were referred to the Mulago Hospital Pediatric Infectious Disease Clinic for further care. The study population and results of primary analysis are described in more detail elsewhere (*6*).

**Figure 1:**
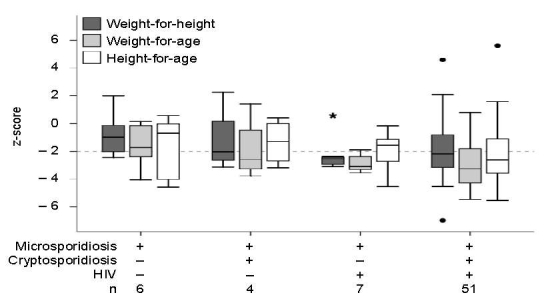
Nutritional status of children in Uganda with microsporidiosis with or without HIV or concurrent cryptosporidiosis. The infection status and number of children in each infection category are shown. Outliers are indicated with dots (1.5–3 interquartile ranges [IQRs]) or the asterisk (>3 IQR). The dashed horizontal line indicates conventional cut-off for malnutrition (z-score <2), horizontal lines in each column indicate the median score, and error bars indicate the highest and lowest z-scores excluding outliers.

A complete set of anthropometric measures (age, weight, height, weight-for-age z-score, height-for-age z-score, and weight-for-height z-score) was available for 224 children. Wasting was twice as likely in children with microsporidiosis than in children without the infection (Table 1). Microsporidiosis was strongly associated with HIV and concurrent cryptosporidiosis. These infections likely compound the poor nutritional status of children with microsporidiosis, although this assessment is limited by sample size (Figure 1).

**Table 1:** Clinical features of 224 children with persistent diarrhea with and without cryptosporidisis, Uganda*

Feature	Total	Microsporidiosis		Crude OR (95% CI)	p value†
		Yes	No		
No. patients	224	68	156		
Age category, mo (SD)					
<6, no. (%)	32 (14.3)	5 (7.4)	27 (17.3)	1.0	
7–12, no. (%)	108 (48.2)	31 (45.6)	77 (49.4)	2.2 (0.8–6.2)	0.137
13–24, no. (%)	69 (30.8)	24 (35.3)	45 (28.8)	2.9 (1.0–8.4)	0.048
>25, no. (%)	14 (6.3)	8 (11.8)	6 (3.8)	7.2 (1.7–29.9)	0.004
Female sex, no. (%)	89 (39.7)	29 (42.6)	60 (38.5)	0.8 (0.5–1.5)	0.556
Nutritional status‡					
Mean WHZ (SD)	–1.44 (1.79)	–1.76 (1.83)	–1.30 (1.76)		0.077
Mean WAZ (SD)	–2.61 (1.41)	–2.76 (1.60)	–2.55 (1.32)		0.356
Mean HAZ (SD)	–2.16 (1.77)	–2.10 (1.90)	–2.19 (1.73)		0.750
Wasted, no. (%)	94 (42.0)	37 (54.4)	57 (36.5)	2.1 (1.2–3.7)	0.013
Underweight, no. (%)	148 (66.1)	47 (69.1)	101 (64.7)	1.2 (0.7–2.2)	0.525
Stunted, no. (%)	121 (54.0)	39 (57.4)	82 (52.6)	1.2 (0.7–2.2)	0.508
Concurrent cryptosporidiosis, no. (%)	63 (28.1)	55 (80.9)	8 (5.1)	78.3 (30.8–199.1)	<0.001
HIV+, no. (%)	77 (34.4)	58 (85.3)	19 (12.2)	41.8 (18.3–95.4)	<0.001
HIV+ and cryptosporidiosis, no. (%)	54 (24.1)	51 (75.0)	3 (1.9)	153.0 (43.1–543.5)	<0.001

*OR, odds ratio; CI, confidence interval. †Continuous variables compared by using 2-sided *t* test. Categorical variables were compared by using Pearson χ^2^ test. ‡WHZ, weight-for-height z-score; WAZ, weight-for-age z-score; HAZ, height-for-age z-score. Children were considered wasted, underweight, or stunted if WHZ, WAZ, or HAZ were <–2.0, respectively.

Linear regression models were used to describe weight and height gain in study children. Weight and height were treated as continuous dependent variables. When regressed on age, the slopes of the curves represent the rate of weight and height gain, respectively. All variables were transformed to the natural logarithmic scale.

For initial data exploration, several simple models were applied with children stratified according to HIV and *Cryptosporidium* spp. status. Within each strata, slope parameters were compared by using *t* tests to identify differences in growth rates between *E*. *bieneusi*–positive and –negative children. Growth rates were reduced in children with microsporidiosis across all HIV and *Cryptosporidium* strata (Table 2). In HIV-positive children, the rate of weight gain was lower in children with microsporidiosis than in those without microsporidiosis (model 1a vs. model 1b), and some evidence showed that this was also true for HIV-negative children (model 1c vs. model 1d). In children concurrently infected with *Cryptosporidium* spp., rates of weight (model 1e vs. model 1f) and height gain (model 2e vs. model 2f) were lower in children with microsporidiosis.

**Table 2:** Rate of weight (model 1) and height gain (model 2) in children with and without microsporidiosis when stratified by HIV and *Cryptosporidium* infection, Uganda*

Model	No.	Infection status				Measurement at age:			R^2^	Rate (95% CI)	Difference†	p value‡
		Micro	Crypto	HIV		12 mo	36 mo	60 mo				
1a	58	+		+		6.63	8.46	9.48	0.27	0.222 (0.13–0.32)	−0.147	0.016
1b	19	–		+		6.69	10.03	12.11	0.73	0.369 (0.25–0.49)		
1c	10	+		–		7.60	8.77	9.37	0.28	0.130 (−0.04–0.30)	−0.177	0.056
1d	137	–		–		6.92	9.70	11.35	0.33	0.307 (0.23–0.38)		
1e	55	+	+			6.65	7.98	8.69	0.17	0.166 (0.07–0.27)	−0.338	<0.001
1f	8	–	+			6.50	11.30	14.62	0.82	0.504 (0.27–0.74)		
1g	13	+	–			7.40	9.50	10.67	0.51	0.227 (0.08–0.37)	−0.081	0.336
1h	148	–	–			6.91	9.69	11.34	0.39	0.308 (0.25–0.37)		
2a	58	+		+		69.55	82.83	89.84	0.63	0.159 (0.13–0.19)	−0.035	0.123
2b	19	–		+		70.46	87.20	96.28	0.82	0.194 (0.15–0.24)		
2c	10	+		–		71.25	81.47	86.70	0.71	0.122 (0.06–0.19)	−0.040	0.206
2d	137	–		–		69.17	82.64	89.77	0.55	0.162 (0.14–0.19)		
2e	55	+	+			69.49	82.03	88.61	0.59	0.151 (0.12–0.19)	−0.085	0.009
2f	8	–	+			68.75	89.10	100.51	0.81	0.236 (0.12–0.35)		
2g	13	+	–			71.49	82.73	88.55	0.75	0.133 (0.08–0.18)	−0.034	0.240
2h	148	–	–			69.41	83.38	90.81	0.61	0.167 (0.15–0.19)		

*For ease of interpretation, predicted measurements at various ages are shown in addition to the rate of growth in ln(kg or cm)/ln(months). Micro, microsporidiosis; Crypto, cryptosporidiosis; CI, confidence interval. †Rate of growth of children with microsporidiosis minus rate of growth of children without microsporidiosis in ln(kg or cm)/ln(months). ‡By *t* test comparing rate of growth of children with and without microsporidiosis.

**Figure 2:**
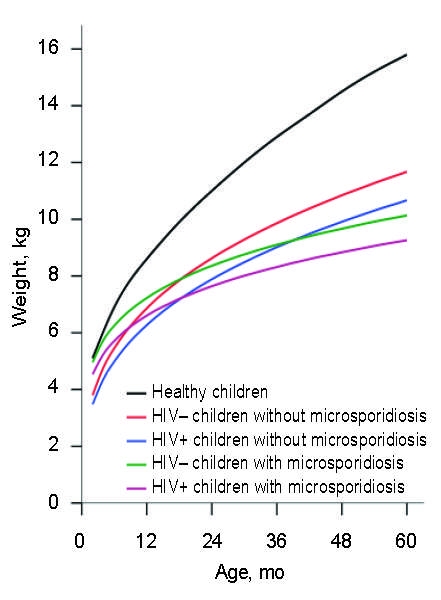
Weight-for-age growth curves of study children (as modeled by multiple linear regression) and reference populations in Uganda (*11*). Curves represent the median weight-for-age, averaged between boys and girls and controlling for concurrent *Cryptosporidium* spp. infection. The difference, 95% confidence interval, and significance of the interaction term between *Enterocytozoon bieneusi* and age reflect the difference in growth rates of children with and without microsporidiosis in ln(kg)/ln(age). R^2^ = 0.42, difference = –0.133, 95% confidence interval –0.23 to –0.03, p = 0.009.

Adjusted growth rate estimates were obtained by fitting a multiple linear regression model that controlled for the effect of sex, HIV status, and concurrent cryptosporidiosis. The independent variable of interest was an interaction term between *E*. *bieneusi* and age, which reflected the difference in the growth rates of children with and without microsporidiosis. Interaction terms between *E*. *bieneusi*, *Cryptosporidium* spp., HIV, and age were also explored but were excluded from the final model because they did not improve model fit. When we simultaneously adjusted for sex, HIV status, and concurrent cryptosporidiosis, rate of weight gain remained significantly lower in children with microsporidiosis (p = 0.014). However, rate of height gain was not significantly different between children with and without microsporidiosis (p = 0.151). Predicted weight-for-age growth curves are shown in Figure 2, which also displays reference curves for healthy Ugandan children (*11*). The growth trajectory of children with microsporidiosis was such that by age 5, these children were predicted to weigh ≈1.3 kg less than children without microsporidiosis. This finding exceeded the predicted difference in weight in children with and without HIV (0.74 kg) at the same age.

## Conclusions

Given consistent reports of severe wasting and malnutrition in HIV-positive adults with microsporidiosis, it is conceivable that *E*. *bieneusi* infection early in life may result in malnutrition. Two cross-sectional studies attempted to correlate microsporidiosis with poor anthropometric status but did not find a significant association (*7**,**12*). However, the dichotomous method used for these assessments is sensitive to the choice of cut-off values. Although children who fall >2 SDs below the reference growth curves are conventionally categorized as malnourished, this cut-off does not denote a biologically meaningful distinction between healthy and malnourished children.

Using regression analysis, we present evidence that microsporidiosis is associated with growth faltering in children in Uganda. In this approach, anthropometric data were treated as continuous variables, thus avoiding arbitrary categorization of children into malnourished and normally nourished groups. Lack of evidence for an independent effect of microsporidiosis on linear growth might be explained by the fact that these children were currently infected with *E*. *bieneusi*, and longer intervals are needed to document decelerating linear growth. In contrast, weight-for-age reflects chronic and acute nutritional states. Because all study participants had a history of persistent diarrhea and stool was generally collected on the day of hospitalization, nosocomial acquisition of microsporidiosis was unlikely.

There are several limitations to this analysis. The causal role of *E*. *bieneusi* in childhood malnutrition cannot be inferred because of the retrospective and cross-sectional study design. Although intestinal infection in children may impair absorption of nutrients (as documented in adults), malnourished children may also have immune defects that predispose them to *E*. *bieneusi* infection. Because the PCR was specific for *E*. *bieneusi*, we cannot rule out that some children were infected with other microsporidian species. However, *E*. *bieneusi* is the more common of 2 species known to cause intestinal microsporidiosis (*13**,**14*). In previous studies at Mulago Hospital, 16.8% of children with acute diarrhea and 16.8% of children without diarrhea had microsporidiosis (*7*). Because all children in the current study had persistent diarrhea, direct comparison between groups was possible without the need to control for diarrhea status. However, we cannot comment on the effect of acute or subclinical infection on nutritional health. Residual confounding may exist through sociodemographic factors not accounted for in the analysis. Sociodemographic data were limited to the accessibility of safe drinking water and type of sanitary facility in the household, neither of which were associated with microsporidiosis (data not shown). Finally, use of cross-sectional data is a major limitation because measurements obtained at a single point in time do not capture individual growth trends. Such data make it difficult to assess the effect of a particular episode of illness on growth attainment (*15*). To this extent, longitudinal anthropometric assessment is the only means of detecting growth faltering that results from *E*. *bieneusi* infection in childhood.

Although our results suggest an association between reduced weight gain and microsporidiosis, further studies are required to determine the role of *E*. *bieneusi* in childhood malnutrition. Longitudinal studies enabling comparison of preinfection and postinfection weights in individual children are needed to establish the direction of causation. It will be particularly useful to identify whether the period of reduced weight gain is followed by catch-up growth. Because microsporidiosis is highly prevalent in children in developing countries, the finding that the infection has a lasting effect on growth would highlight the importance of nutritional rehabilitation and provide impetus to develop therapeutics suitable for use in young children.
